# Numerical Simulation of Fire in Underground Commercial Street

**DOI:** 10.1155/2022/4699471

**Published:** 2022-09-13

**Authors:** Hao-Wei Yao, Ke-Feng Lv, You-Xin Li, Jin-Guang Zhang, Zhong-Bin Lv, Dong Wang, Zhen-Yu Zhan, Xiao-Ge Wei, Huai-Tao Song, Heng-Jie Qin

**Affiliations:** ^1^College of Building Environment Engineering, Zhengzhou University of Light Industry, Zhengzhou 450001, China; ^2^Zhengzhou Key Laboratory of Electric Power Fire Safety, Zhengzhou 450001, China; ^3^State Grid Henan Electric Power Research Institute, Zhengzhou 450052, China

## Abstract

In this study, while aiming at the prevention of fire accidents in underground commercial streets, an underground commercial street is selected as a research object, and the building fire is numerically simulated using the PyroSim software. Fire simulation scenarios are divided according to different fire zones by analyzing the temperature, carbon monoxide (CO) concentration, and visibility in the smoke layer inside a building. The available safe evacuation time is calculated according to the critical fire hazard judgment conditions. We found that the time when the flue gas temperature and CO concentration reached the critical value in the fire site was longer than the time when the visibility reached the critical value reducing or even avoiding the spread of smoke from the fire area to the evacuation stairs can provide effective help for crowd evacuation. Finally, the safety of the building is evaluated, and fire prevention countermeasures are defined based on the actual situation and fire numerical simulation results to reduce fire incidence, casualties, and economic losses.

## 1. Introduction

In recent years, the economic construction work all over China has shown a vigorous and upward development trend, which has led to the rapid reduction in available land resources and cost increase of the ordinary business model [1, 2]. To alleviate the economic pressure brought by limited land resources, underground commercial streets have developed rapidly. Underground commercial streets have been developing rapidly [3], introducing many potential safety hazards threatening people's safety and causing enormous economic losses.

Due to the complex structure, closed space, narrow and long streets, much electrical equipment and floating personnel, and many dense materials, there has been a high risk of fire in the underground commercial streets [4]. The fire has the characteristics of a high probability of occurrence, a large amount of smoke, fast fire spread, easy flashover, difficult personnel evacuation, poor explosion discharge capacity, and difficult fire-fighting, which severely threatens the safety of people's lives and property. Therefore, by using scientific methods to analyze the law of fire in underground commercial streets and understand the evacuation rules of personnel in the case of a fire, economic losses and casualties could be greatly reduced [5].

Therefore, the fire simulation evaluation and analysis of underground buildings have been very important. This can not only ensure people's safety and reduce economic losses but also prevent the occurrence of fire accidents. The development of computer simulation technology allowed its application in fire simulation and personnel evacuation tests in recent decades. The implementation of computer simulation technologies in fire analysis had also been used in the world, and great achievements have been achieved.

Roman et al. [6] use the “fire dynamics simulator” (FDS) software to simulate the fire spread on the surface of the across facade system and compare the experimental data with the calculated data to improve some unknown factors that may occur in the process of fire simulation. Bystrom et al. [7] introduced the reliability analysis to the fire tests in different scenarios and performed the simulation analysis to analyze the similarities and differences between fires in real situations and computer simulations. Anderson et al. [8] investigate a traveling fire scenario in an elongated structure by FDS software. The fire spread is controlled manually by initiating fires consecutively in the pools. The post-test simulations indicate that although there are a lot of variations not included in the model similar results were obtained as in the test.

At present, there have been dozens of fire simulation software. Most of them establish standard fire models, such as TAKAHASHIS and VEGAS, that perform three-dimensional (3D) motion graphics on the basis of building entities. Based on the FDS fire simulation software, the NIST Research Institute has provided a graphical user interface and developed a more functional fire simulation software PyroSim to evaluate the fire safety of buildings [9].

Yue et al. [10] used the regional fire simulation method to simulate the fire smoke. According to the height of the smoke layer, they compared and calculated the time required for safe evacuation and the arrival time of danger. They also quantitatively predict and evaluate the fire evacuation safety of underground commercial streets. Zeng et al. [11] developed a model of extended cellular automata, in which the concepts of “risk” and “vision” have been introduced. Wu et al. [12] used variable fire source power to conduct fire experiments on a long channel. They studied the installation of the ceiling in underground commercial streets and obtained the flow characteristics of the fire smoke under different air supplement and smoke exhaust conditions. Li et al. [13] implemented a fusion digital simulation (FDS) model for a demonstration section of the cable corridor of the urban utility tunnel and analyzed the performance of the water mist fire extinguishing system with low, medium, and high operating pressures under different spatial structures, fire source positions, and ambient air velocities. Zhou et al. [14] used the Navier Stokes equation to simulate the real combustion process and found the capability of the physics-based fire model in representing some features of real flames. The proposed quantitative analysis of virtual flames serves to evaluate the similarity between a virtual and a real fire. Deng et al. [15] used FDS software to simulate the spread of fire smoke in staircases with different structures and found that staircases without stairwell structures are more conducive to safe evacuation.

However, in most studies, there were few numerical simulation studies on the special occasion of underground commercial streets. In this study, the underground floor of an underground commercial street is selected, and the model and numerical simulation are established through PyroSim.

## 2. Fire Characteristics of Underground Commercial Street

An underground commercial street has the characteristics of both aboveground commercial buildings and underground buildings. Namely, it represents a special architectural form. An underground commercial street has complex functions, includes much electrical lighting equipment, and is characterized by a large space, many combustibles, and high fire probability, which makes it different from other civil buildings [16]. There four main fire characteristics are explained in the following:

### 2.1. High Probability of Fire

As mentioned, the structure and functions of underground commercial streets are complex, while the probability of fire is high. Underground buildings have no natural lighting, so they heavily rely on electrical lighting. Also, the ventilation system requires a certain amount of electric energy. If the power supply line is short-circuited, fire can appear. In addition, clothes, shoes, bags, and electrical equipment used by various shops can greatly increase the fire load of an underground commercial street.

### 2.2. Large Fire Smoke

The generation of fire is accompanied by a large amount of smoke, which is difficult to be eliminated in an underground commercial street environment. The smoke and dissipate heat can be removed from the underground commercial street environment only through the inlet and outlet to the outside and the installed smoke exhaust system. Thus, the ventilation conditions in the underground commercial street environment are relatively poor [17]. Compared with aboveground buildings, a large number of substances in underground commercial streets can produce smoldering due to a lack of oxygen when they are on fire. It should be noted that smoke and harmful gases are difficult to be eliminated, and a large amount of smoke and temperature can gather in the underground commercial street environment [18].

### 2.3. Rapid Fire Spreading

Underground commercial streets mainly include different types of shops, operating with various types of combustible materials, such as clothes, shoes, and leather bags. Therefore, the fire load density is large, which is conducive to fire accidents. As the roads in underground commercial streets are crowded, and the fire spreads rapidly, flashover can easily occur with the continuous increase in temperature [19].

### 2.4. Difficult Evacuation

Compared with the aboveground buildings, the building structures in the underground commercial streets are more complex, and the emergency exit is relatively hidden. Therefore, for people who are not familiar with this type of building structure, it could be extremely difficult to evacuate from the underground commercial streets in the case of a fire. In addition, in the case of a fire, the concentration of smoke continuously increases while the visibility decreases. Hence, there is a great chance that the light of emergency lighting cannot provide visible guidance to people. The related research has shown that when the visibility is reduced to 4 m, the personnel cannot evacuate [20].

## 3. Fire Numerical Simulation Model

### 3.1. Theoretical Basis

The numerical model was established by using PyroSim software, and the fire development under different working conditions was calculated by FDS fire dynamics software. According to FDS technical guidelines [21], the governing equation is expressed as follows:

The equation of the mass conservation is mathematically expressed as follows:

(1) $$\begin{array}{c} \frac{\partial \rho }{\partial t}+\nabla ∙\rho u=m_{b}^{\prime \prime \prime }\text{.} \end{array}$$

The momentum conservation equation is expressed as follows:

(2) $$\begin{array}{c} \frac{\partial }{\partial t}\left(\rho u\right)+\nabla ∙\rho uu+\nabla p=\rho g+f+\nabla ∙\tau_{ij}\text{.} \end{array}$$

u=(*u*, *v*, *w*)^*T*^, ∇=(*∂*/*∂x*, *∂*/*∂y*, *∂*/*∂z*) , They are vectorization operator, and is calculated by the following formula:

(3) $$\begin{array}{c} \begin{array}{c} \tau_{ij}=\mu \left(2S_{ij}-\frac{2}{3}\delta_{ij}\left(\nabla ∙u\right)\right)\\ \delta_{ij}=\left\{\begin{array}{c} 1,i=j\\ 0,i\neq j \end{array}\right.\\ S_{ij}=\frac{1}{2}\left(\frac{\partial u_{i}}{\partial x_{j}}+\frac{\partial u_{j}}{\partial x_{i}}\right),i,j=1,2,3 \end{array}\text{.} \end{array}$$

The energy conservation equation is described as follows:

(4) $$\begin{array}{c} \frac{\partial }{\partial t}\left(\rho h_{s}\right)+\nabla ∙\rho h_{s}u=\frac{Dp}{Dt}+{\dot{q}}^{\prime \prime \prime }-{\dot{q}}_{b}^{\prime \prime \prime }-\nabla ∙{\dot{q}}^{\prime \prime }+\varepsilon \text{,} \end{array}$$

(5) $$\begin{array}{c} \frac{Dp}{Dt}=\frac{\partial p}{\partial t}+u∙\nabla p\text{,} \end{array}$$

(6) $$\begin{array}{c} \varepsilon =\tau_{ij}∙\nabla u\text{,} \end{array}$$

(7) $$\begin{array}{c} q^{\prime \prime }=-k\nabla T-\underset{\alpha }{\sum }h_{s,\alpha }\rho D_{\alpha }\nabla Y_{\alpha }+q_{\gamma }^{\prime \prime }\text{.} \end{array}$$

Equation of state is described as follows:

(8) $$\begin{array}{c} {\bar{P}}_{m}\left(z,t\right)=\rho TR\underset{\alpha }{\sum }\frac{Y_{\alpha }}{W_{\alpha }}=\frac{\rho RT}{\bar{W}}\text{.} \end{array}$$

### 3.2. Model Selection

**Figure 1:**
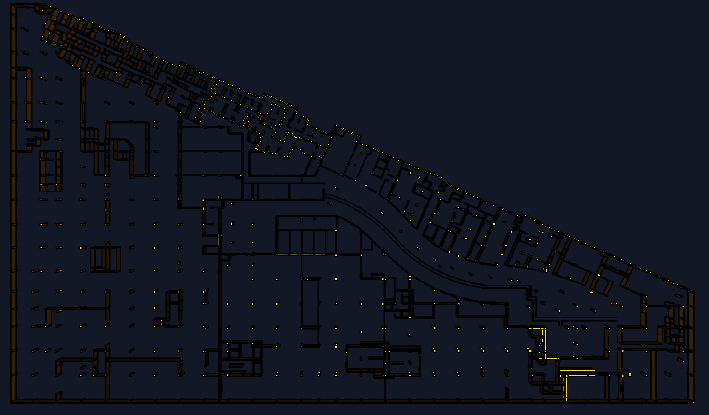
The ground floor plan.

**Figure 2:**
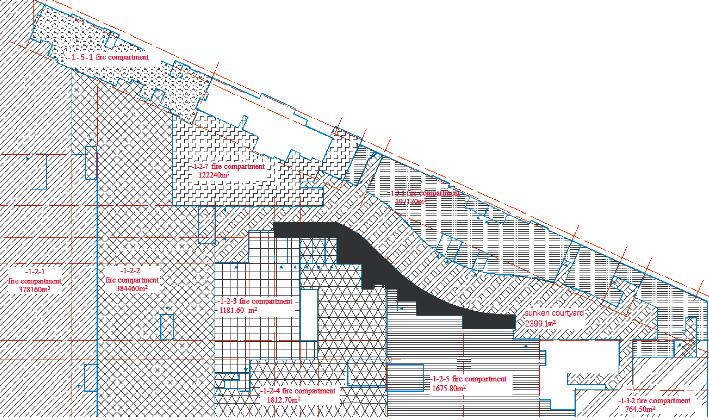
Schematic diagram of the fire compartment.

The research object of this study is the first floor of the underground commercial street in Henan province, and its model is established using the PyroSim simulation software. The main functions of the basement are as follows: there are shops on both sides of the sunken square, supermarkets on the east side of the underground garage, and equipment rooms and motor garages on the other side. With a total construction area of 21,138.28 m^2^, it belongs to the category of large underground commercial streets. The atrium part is connected with the outdoor, as shown in the plan of the first-floor underground presented in Figure 1. Since the building area of the underground building is too large, it was divided into ten fire compartments in the simulations, as shown in Figure 2 Combined with the actual situation, this study performed simulation calculations only for five fire compartments.

### 3.3. Mesh Division

When using the PyroSim software for simulation calculation, it is necessary to divide the grid. The finer the grid is, the smaller the calculation error will be, but much calculation time will be needed. In this study, after several simulations and analyses, the grid parameters of fire scenes H2, H4, and H5 were approximately set to 0.5 m × 0.5 m × 0.5 m [22]. The grid of large fire scenes H1 and H3 was approximately set to 1.0 m × 1.0 m × 1.0 m.

## 4. Fire Scene Simulations

### 4.1. Design Principle of Fire Scene

When studying the fire development process, the fire scene is generally considered to be under the condition of the greatest fire hazard according to the actual situation of a building. The ground floor of an underground commercial street mainly consists of the parking lot, business area, and various machine rooms. All machine rooms are typically located on the ground floor. The electric power circuit is relatively complex, and the power demand is high. Thus, in the case of a fire, it will be very difficult to rescue. In addition, it should be noted that there are many human-related factors that can cause fire, such as littered cigarette butts and intentional malignant events [23].

Because the area of an underground commercial street is large, considering the error between the experimental simulation results and the actual situation is very important. When designing the fire scene, the fire scene simulation has been commonly carried out according to the fire compartment of a considered underground commercial street. At the same time, it has been assumed that the fire appears under the fire control condition of no spraying and no smoke exhaust.

### 4.2. Fire Simulation Scenario Setting

In computer simulations, the maximum heat release rate of a fire source was considered, and it was determined based on statistical data [4]. As shown in Table 1, it has been assumed that the fire appears under the fire control condition of no spraying and no smoke exhaust. This study considered the underground commercial street without sprinkler equipment, so the maximum heat release rate of the fire source was 6 MW. The type of fire source was *T* square fire.

**Figure 3:**
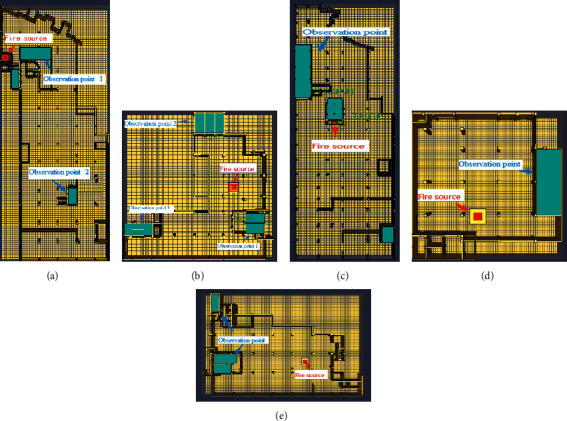
Schematic diagram of fire source locations in a different scenario: H1–H5. (a) Schematic diagram of fire source location H1; (b) schematic diagram of fire source location H2; (c) schematic diagram of fire source location H3; (d) schematic diagram of fire source location H4; and (e) schematic diagram of fire source location H5.

The office area was divided into five fire simulation scenarios according to fire zones, and five of them were simulated according to the fire zones in Figure 2, including 1-2-2, 1-2-4, 1-2-1, 1-2-3, and 1-2-5, which were recorded as fire scenario H1–H5, respectively. The fire locations of different fire areas are shown in Table 2 and Figure 3.

By calculating the area of each burner, the heat release rates of the fire sources in the simulation process were obtained, as shown in Table 3.

Relevant parameters in the PyroSim software were set and only needed to be modified on this basis. According to the underground commercial street considered in this study, the following relevant parameters were set for simulation:

- Initial environment temperature: 20°C
- Initial relative humidity of environment: 50%
- Atmospheric pressure: 101,325 Pa
- Wind speed: zero
- Fire simulation running time: 360 s
- Fire growth type: fast fire
- Floor material: insulating material, nonconductive
- Fire-fighting conditions: no sprinkler, smoke exhaust, and detector.
- Combustion type: electrical appliances, cables, and daily necessities
- Building height: 5.7 m

### 4.3. Fire Hazard Parameter Analysis

The threat degree of fire to personal safety was judged based on the analysis of flue gas temperature, CO concentration, and visibility at 2 m. According to the literature review, the critical safety time in this study should meet two critical conditions, namely, visibility of 10 m and a temperature of 60°C. Also, the concentration of CO should not be higher than 500 ppm (0.05%) [23]. Fire scenarios were divided into two groups based on similarity in size and shape: fire scenarios H1 and H3 were grouped because they were approximately rectangular in shape; fire scenarios H2, H4, and H5 were grouped because they were all approximately square in shape and approximately equal in size; and the smoke temperature, carbon monoxide concentration, and visibility at each evacuation staircase were analyzed and discussed for fire scenarios H1 and H2. Finally, the simulation conditions of five scenarios were obtained.

#### 4.3.1. Flue Gas Temperature Analysis

**Figure 4:**
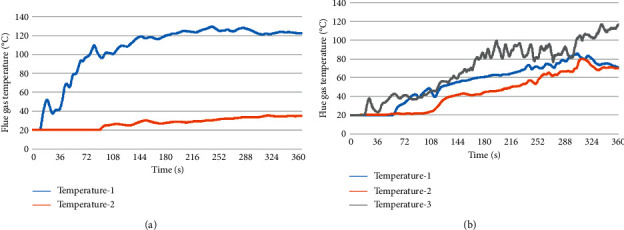
Variations in the smoke temperature with time at the evacuation stairs in different fire scenarios. (a) Fire scenario H1and (b) fire scenario H2.

The change in the flue gas temperature with time at the evacuation staircase in different fire scenarios with a height of 2 m from the ground when the fire source with a power of 6 MW was burning under different fire scenarios is presented in Figure 4.

In fire scenario H1, as shown by curve I in Figure 4(a), the flue gas temperature at evacuation staircase 1 began to increase at 10 s, the critical safety temperature reached 60°C in 50 s; the maximum temperature reaches 129°C at 240 s. As shown by curve II in Figure 4(a), the flue gas temperature at evacuation staircase 2 was equal to room temperature 20°C before 90 s. This was because staircase 2 is far away from the fire source, and the transfer of heat radiation takes time. Then, it began to increase gradually, but the flue gas temperature did not reach the critical safety temperature of 60°C within 300 s.

In fire scenario H2, as shown by curve I in Figure 4(b), the flue gas temperature at evacuation staircase 1 began to increase gradually at 60 s, and the temperature reached a critical state at 175 s. Finally, the maximum temperature was 85°C at 300 s. As shown by curve II in Figure 4(b), due to heat transfer [24], although the flue gas did not reach evacuation staircase 2 before 100 s, the temperature was rising continuously. After 100 s, the temperature increase rate began to increase and reached the critical safety temperature at 255 s. As can be seen from curve III in Figure 4(b), at 25 s, the temperature decreased rapidly from 38°C to 22°C. This was due to the short temperature drop caused by the slow diffusion of the high-temperature flue gas toward both sides after reaching evacuation stairs 3. With time, the flue gas temperature at evacuation staircase 3 reached the critical safety temperature at 140 s and continued to increase.

#### 4.3.2. Carbon Monoxide Concentration Analysis

**Figure 5:**
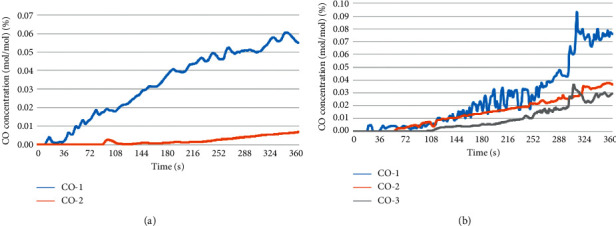
Variations in the CO concentration with time at evacuation stairs in different fire scenarios. (a) Fire scenario H1and (b) fire scenario H2.

The change in CO concentration with time at the evacuation staircase in different fire scenarios at the height of 2 m from the ground is presented in Figure 5. When the concentration of CO in the flue gas was higher than 500 ppm (0.05%), if people were in the flue gas area, their life safety would be threatened.

In fire scenario H1, as shown by curve 1 in Figure 5(a), at the beginning of the fire, the CO concentration suddenly increased and then rapidly decreased, which was due to the short increase and fall in the smoke after reaching this point and spreading along the roof to the other places. As shown in Figure 5(a), the CO concentration reached the critical safety concentration at 240 s. Furthermore, as shown by curve II in Figure 5(a), due to the large area of the fire compartment, the evacuation staircase at the bottom of the fire compartment had no change in the CO concentration before 90 s, and the CO concentration continued to increase after 90 s. The critical safe CO concentration of 0.5% was not reached in the simulation process.

In the fire scenario H2, as shown by curve I in Figure 5(b), in the whole simulation process (360 s), the CO concentration constantly increased, reaching the maximum value at about 290 s, and the CO concentration reached the near safe CO concentration in 290 s. As shown by curve II in Figure 5(b), the CO concentration started to increase at 55 s, and the increase rate reached the maximum at approximately 290 s, but the maximum CO concentration was less than the critical safe CO concentration in the whole simulation process. Furthermore, as shown by curve 3 in Figure 5(b), the CO concentration began to increase at 100 s, and the increase rate reached the maximum at about 290 s, but the maximum CO concentration was less than the critical safe CO concentration in the whole simulation process.

The comparison of the results is presented in the three curves in Figure 5(b) shows that the CO concentration increased rapidly at about 290 s, indicating that the fire was in the stage of violent combustion, and the combustion was the most vigorous. Thus, people should escape safely before this phase; otherwise, their lives will be in danger [25].

#### 4.3.3. Visibility Analysis

**Figure 6:**
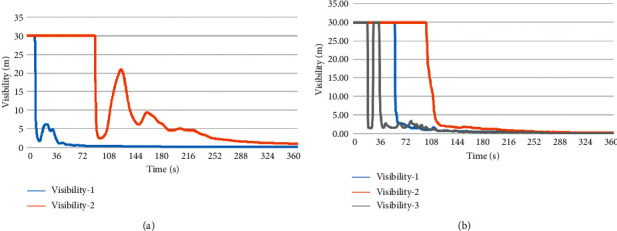
Variations in visibility with time at evacuation stairs in different fire scenarios. (a) Fire scenario H1and (b) fire scenario H2.

Figure 6 shows the change in visibility with time at the evacuation stairs in different fire scenarios with a height of 2 m from the ground when the fire source with a power of 6 MW was burning under different fire scenarios. When the visibility of the environment was reduced to 10 m, it was very easy for people to lose their direction and find no emergency exit. Especially for those who are not familiar with the building structure, their life safety will be threatened.

In fire scenario H1, as shown by curve 1 in Figure 6(a), the distance between the evacuation staircase and the fire occurrence point was only 5 m, and the visibility was rapidly reduced to 2 m in 15 s. However, since the point was located at the corner of fire scenario H1, the available safe evacuation time was longer than 15 s. As shown by curve II in Figure 6(a), the visibility at this point was 30 m before 98 s and then decreased rapidly. The visibility stabilized below 10 m in 150 s, so the available safe evacuation time was 140 s.

In the fire scenario H2, as shown by curve 1 in Figure 6(b), since evacuation staircase 1 was far away from the fire source and was located at the edge of the fire compartment, the visibility at this point was 30 m before 60 s, but after 60 s, the visibility dropped rapidly to 3 m and finally reduced to zero. Therefore, the available safe evacuation time of the evacuation staircase was 60 s. As shown by curve II in Figure 6(b), although the evacuation staircase was close to the fire source, since the entrance of the staircase was in the center of the fire compartment, the risk degree of this point was less than that of the point on evacuation staircase 1. According to the jet effect at the top of the flue gas [23], the visibility was 30 m before 105 s, and then rapidly decreased to 3 m. The time to reach 10 m visibility at this point was about 110 s, so the available safe evacuation time of this evacuation staircase was 110 s. As shown by curve 3 in Figure 6(b), the smoke was blocked by the wall and diffused around after reaching evacuation staircase 3, resulting in a sudden drop in visibility at 25 s. Then, at the time of 40 s, the visibility was as low as 2 m, so the available safe evacuation time was 40 s.

### 4.4. Results Analysis

Through the simulation of the fire development process, the statistical data of the smoke temperature, CO concentration, and visibility were obtained, and the available safe evacuation time of the fire compartment was judged by analyzing the data image so as to ensure the life and property safety of personnel.

The fire simulation process of the five scenarios was analyzed in turn, and the data on the five simulations were summarized to obtain the critical safety time of different evacuation stairs under each fire scenario, as shown in Table 4. According to the simulated data, the time when the flue gas temperature reached the critical value in the fire site was longer than the time when the CO concentration reached the critical value. The time when the flue gas temperature and CO concentration reached the critical value in the fire site was longer than the time when the visibility reached the critical value. In addition, the results indicated that the smoke was the most direct affecting factor of the safe evacuation. A large amount of smoke would make people fear, thus losing their ability of judgment and increasing the sense of panic. Therefore, the judgment basis of safe evacuation should be visibility [26].

### 4.5. Discussion

The generality of the results depends on the changes in different parameters. In this study, we only focus on the changes in flue gas temperature, CO concentration, and visibility at specific points without spraying in the underground commercial street. We must make it clear that our research was not to obtain general conclusions but to verify verified schemes that can be used to further build more complex models.

## 5. Conclusion

This paper studies the smoke flow in the underground commercial street in the case of a fire. The theoretical knowledge about fire dynamics is used to simulate the dynamic process of fire development using the PyroSim computer simulation software.

Combined with the actual situation of a building, the fire scene is defined according to the most dangerous fire situation, and the smoke flow is simulated in the case of a fire using the PyroSim fire simulation software. The critical time of safe evacuation is determined according to the critical conditions of the flue gas temperature, visibility, and CO concentration. This time represents the available safe evacuation time. This study points out the hidden fire hazards of an underground commercial street and defines certain recommendations for fire safety.

The specific conclusions of this work can be summarized as follows:

- The flow of fire smoke in buildings is a complex problem. The fire scenario simulation by the PyroSim simulation software basically conforms to the diffusion mechanism of smoke in buildings.
- According to the PyroSim fire simulation and simulation data analysis, in the case of a fire, the most affecting factor is the lack of visibility caused by smoke. Therefore, reducing or even avoiding the spread of smoke from the fire area to the evacuation stairs can provide effective help for crowd evacuation.
- According to the PyroSim fire simulation and simulation data analysis, in the case of a fire, the most affecting factor is the lack of visibility caused by smoke. Therefore, reducing or even avoiding the spread of smoke from the fire area to the evacuation stairs can provide effective help for crowd evacuation.
- The presented results can be used as a reference to improve the fire-fighting systems of the underground commercial street, form effective protection, prevent the fire from entering the initial growth stage, prevent the wanton spread of smoke, and protect people's safety. In addition, this study indicates that the skills of fire-fighting staff should be improved through regular fire safety education and training so as not to be at a loss in case of a fire in the future.

In addition, in this simulation analysis of an underground commercial street fire, the optimal safe evacuation time was obtained through computer simulation. We will also conduct real evacuation experiments in the future, so as to obtain a safe evacuation time and determine whether the underground commercial street is safe, so there were still many problems that need to be further studied.

## Figures and Tables

**Table 1:** The maximum heat release rate.

Place	Maximum heat release rate *Q* (MW) with spraying equipment	Maximum heat release rate *Q* (MW) without spraying equipment
Commercial street	5	6
Public place	2.5	8
Garage	1.5	3
Warehouse	4	20
Stalls	1	4
Office area	1.5	6

**Table 2:** The fire location in different fire scenarios.

Fire compartment	Fire scene	Fire location
1-2-2	H1	Supply fan room
1-2-4	H2	Electrical equipment in the meat area of a supermarket
1-2-1	H3	Electrical room at the center of the 1-2-1
1-2-3	H4	Cash register in the fresh- and cooked-food areas in a supermarket
1-2-5	H5	Air-conditioner engine room

**Table 3:** The fire heat release rate in different fire scenarios.

Fire compartment	Fire scene	Heat release rate of fire source (kW/m^2^)
1-2-2	H1	6,000
1-2-4	H2	1,500
1-2-1	H3	2,500
1-2-3	H4	1,500
1-2-5	H5	2,343.75

**Table 4:** Summary of critical safety time.

Fire scene	Evacuation staircase number	Temperature safety time (s)	CO concentration safety time (s)	Visibility safety time (s)	Available safety evacuation time (s)
H1	1	50	240	15	15
	2	>360	>360	140	

H2	1	175	290	60	40
	2	255	>360	110	
	3	140	>360	40	

H3	1	170	185	90	65
	2	85	120	65	

H4	1	>360	>360	185	130
	2	>360	240	130	

H5	1	170	175	65	65
	2	>360	>360	>360	

## Data Availability

The data of the study can be provided by contacting the corresponding author.
